# Optogenetic Inhibition of the Orbitofrontal Cortex Disrupts Inhibitory Control during Stop-Change Performance in Male Rats

**DOI:** 10.1523/ENEURO.0015-24.2024

**Published:** 2024-05-10

**Authors:** Adam T. Brockett, Neeraj Kumar, Paul Sharalla, Matthew R. Roesch

**Affiliations:** ^1^Department of Psychology, University of Maryland, College Park, Maryland 20742; ^2^Program in Neuroscience and Cognitive Science, University of Maryland, College Park, Maryland 20742; ^3^Department of Biological Sciences, University of New Hampshire, Durham, New Hampshire 03824

**Keywords:** inhibitory control, OFC, optogenetics, response inhibition, stop-signal

## Abstract

Historically, the orbitofrontal cortex (OFC) has been implicated in a variety of behaviors ranging from reversal learning and inhibitory control to more complex representations of reward value and task space. While modern interpretations of the OFC's function have focused on a role in outcome evaluation, these cognitive processes often require an organism to inhibit a maladaptive response or strategy. Single-unit recordings from the OFC in rats performing a stop-change task show that the OFC responds strongly to STOP trials. To investigate the role that the OFC plays in stop-change performance, we expressed halorhodopsin (eNpHR3.0) in excitatory neurons in the OFC and tested rats on the stop-change task. Previous work suggests that the OFC differentiates between STOP trials based on trial sequence (i.e., gS trials: STOP trials preceded by a GO vs sS trials: STOP trials preceded by a STOP). We found that yellow light activation of the eNpHR3.0-expressing neurons significantly decreased accuracy only on STOP trials that followed GO trials (gS trials). Further, optogenetic inhibition of the OFC speeded reaction times on error trials. This suggests that the OFC plays a role in inhibitory control processes and that this role needs to be accounted for in modern interpretations of OFC function.

## Significance Statement

The orbitofrontal cortex (OFC) is a highly interconnected brain region thought to be essential for healthy cognitive function. Historically, research has implicated the OFC in the inhibition of inappropriate actions; however, more recent work has focused on its role in guiding value-based decision-making. Using a modified version of a canonical inhibitory control task in combination with optogenetics, we show that while important for value-based decision making, disruption of excitatory neurons in the OFC impairs inhibitory control processes as well. This highlights the need for theoretical accounts of OFC function to accommodate its role in both types of cognitive processes.

## Introduction

The ability to make decisions about what to approach and what to avoid is an essential component of cognitive health. Navigating these critical decisions requires an organism to maintain an accurate understanding of its needs and internal state as well as a knowledge of behaviors or action that can help it to achieve its goals. This mapping between goal and action is heavily influenced by previous experience. Actions that lead to success are often more likely to be repeated than those that have led to failure. Understanding how the brain converts this varied array of variables into a single response has emerged as a major focus of modern systems neuroscience.

Almost from the moment the complexity of this problem was fully articulated, neuroscientists began zeroing in on the orbitofrontal cortex (OFC) as one of the primary candidates for facilitating the conversion of goals to actions (Bechara et al., 2000; Schoenbaum et al., 2009; Wilson et al., 2014; Rudebeck and Rich, 2018; Brockett and Roesch, 2021). The OFC is a highly interconnected swath of cortex located just behind the eyes. Early research into its function was inspired in part by the case studies of Phineas Gage and others, in which either an accident or surgical intervention damaged the OFC. Patients exhibited an inability to inhibit inappropriate responses (Harlow, 1993; Schoenbaum et al., 2009; Van Horn et al., 2012). Further work showed that aspiration lesions made to the OFC of primates disrupted reversal learning (Izquierdo and Murray, 2004); however, later work showed that excitotoxic lesions to primate OFC, which spared fibers of passage, did not (Rudebeck et al., 2013). Other behavioral and single-unit recording studies soon began, suggesting that neural activity in the OFC better reflects expectations about future outcomes rather than response inhibition directly (Schultz et al., 2000; Kheramin et al., 2003; McAlonan and Brown, 2003; Schoenbaum et al., 2003; Winstanley et al., 2004; Chudasama et al., 2007; Ghods-Sharifi et al., 2008; Swick et al., 2008; Padoa-Schioppa, 2011). As scrutiny of the OFC grew, work focusing directly on the role of the OFC in response acquisition began describing a kind of map whereby neuron OFC appeared to track goal, actions, and outcomes, thereby guiding and optimizing future decision-making (Schoenbaum et al., 2009; Wilson et al., 2014). These revised accounts of OFC function generally view earlier reports of difficulties in response inhibition as a part of mismatch in an individual's ability to map the correct outcome onto the appropriate action (Schoenbaum et al., 2009). Complicating this interpretation is the fact that the behavioral tasks used often that supported these conclusions were designed specifically to examine the relationship between response and outcome.

While the OFC does clearly support outcome evaluation, several pieces of data in rodents (Chudasama and Robbins, 2003; Eagle et al., 2008; Bari et al., 2011; Bryden and Roesch, 2015), nonhuman primates (Balasubramani et al., 2020), and humans (Solbakk et al., 2014) in the context of canonical inhibitory control paradigms still implicate the OFC in inhibitory control processes as well. Critically, unlike reward outcome paradigms that often require a remapping of response outcome contingencies, inhibitory control paradigms, such as the stop-signal task, explicitly pit two responses against one another with reward being equal (Verbruggen et al., 2019). Understanding the extent to which the OFC supports inhibitory control is essential to our understanding of this critical brain area that supports almost all aspects of cognition.

To further examine the OFC's involvement in inhibitory control, we expressed halorhodopsin (eNPHR3.0) in excitatory neurons in the OFC and then tested rats on a stop-change task. We found that optogenetic inhibition of excitatory neurons impaired performance on STOP trials that were preceded by GO trials and speeded reaction time (RT) on errors. Collectively, these findings suggest a role for the OFC in inhibitory control and highlight a need for future research into the function of the OFC to account for this possibility.

## Materials and Methods

### Animals

Seven male Long–Evans rats (*n *= 7; weight at arrival: 175–200 g) were obtained from Charles River Laboratories. Rats were housed on a 12/12 h light/dark schedule with lights on at 6:00 A.M. EST. All training, behavioral testing, and recordings occurred between 11:00 A.M. and 6:00 P.M. EST. This study was approved by the Institutional Animal Care and Use Committee and conformed to the National Research Council guidelines (National Research Council (US) Committee for the Update of the Guide for the Care and Use of Laboratory Animals, 2011).

### Stop-change task

**Figure 1.**
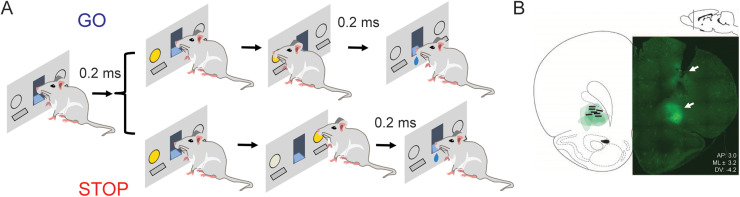
Task design and behavioral analysis. ***A***, Schematic of stop-change task. Following the house lights, rats made a nose poke for 200 ms before a light cue was illuminated on either the right or left side. On 80% of trials (GO trials), this light corresponded to the correct direction that rat needed to move in order to receive reward. On 20% of trials, a second light was illuminated after the initial GO cue directing the rat to inhibit their initial response to the first cue in favor of making a response in the direction of the second cue. ***B***, Representative image of optical fiber tract and EYFP expression in OFC. Left: Green contours represent area of expression for each animal. Black lines represent optical fiber placement for each animal. Right: Example EYFP expression in the OFC. Top white arrow indicates fiber tract; bottom white arrow indicates expression.

Behavioral testing was conducted in modular chambers 10″ × 12″ × 11.75″ (Med Associates). On one wall of the chamber, a fluid well/center port was located just above two adjacent levers. One light was located above each lever, and a house light was located above the fluid well. An illustration of the response panel is provided in Figure 1*A*. Task control was implemented via a computer using a custom script written in the MedPC language. Lever presses and well entry times were monitored by disruption of photobeams.

The trial design is illustrated in Figure 1*A*. Each trial began with illumination of the house light that instructed the rat to make a nose poke into the fluid well. Nose poking initiated a 200 ms precue delay period. If the rat exited the port during this 200 ms period, the trial was aborted, and house lights were extinguished. On 80% of trials (i.e., GO trials), nose poking led to the illumination of the left or right light, which signaled the lever (i.e., left or right) the rat needed to press. After making its selection, the rat then needed to re-enter the center fluid well and hold for 200 ms before receiving a small liquid sucrose reward (∼70 µl of cold 10% sucrose solution). On the remaining 20% of trials (i.e., STOP trials), nose poking led to the illumination of either the left or right light, which was then extinguished, and the light over the opposite lever was then illuminated after a stop-signal delay (350–1,000 ms). This second light remained illuminated until a lever press was made. On STOP trials, rats were required to stop the movement signaled by the first light and respond in the direction of the second light. Upon pressing the lever signaled by the second light, the rat then needed to re-enter the center fluid well and hold for 200 ms before receiving a small liquid sucrose reward (∼70 µl 10% sucrose solution). GO and STOP trials were randomly interleaved. Error trials (incorrect press) were immediately followed by the extinction of house lights and ITI onset of 4 s. Rats had to exit the central well before the next trial began (i.e., illumination of house lights). Trials were presented in a pseudorandom sequence such that left and right trials were presented in equal numbers.

### Surgical procedures

Rats were trained on the stop-change task for ∼1 month before undergoing virus injection and ferrule implantation surgery. Training consisted of 7 d of shaping nosepoke and lever press behaviors, before rats were exposed to a GO-only version of the stop-change task for an additional 1.5 weeks. In the final 1.5 weeks of training, STOP-change trials were gradually introduced first at a ratio of 1/20, then 2/20, and 3/20 (STOP-change/GO trials) per session before reaching the final ratio of 4/20. Rats were allowed to practice on the final stop-change task for 5 d before surgery and had to demonstrate an ability to complete a minimum of 150 trials at least once during this 5-d window. All surgical procedures followed guidelines for aseptic technique and were conducted in a dual arm rat stereotax (David Kopf Instruments) fit with a single microsyringe pump driver (WPI). All rats received a bilateral injection of rAAV5-CaMKIIa-eNPHR3.0-EYFP-WPRE-PA (UNC Vector Core; titer: 5.7 × 10^12^ particles/nl), 0.75 µl per hemisphere, targeting the OFC (coordinates: AP, +3.0 mm; ML, ±3.2 mm; DV, −4.2 mm from the bregma). All injections were performed with a 10 µl microsyringe with a blunt 33 ga needle (WPI) held at a 10° angle. Following injection, the needle sat undisturbed for 5–10 min before being slowly removed. OFC coordinates were chosen based on previously published single-unit recording studies targeting the same area (Bryden and Roesch, 2015). Immediately following injection, 5.0 mm steel FC ferrules (Thorlabs) were lowered to the same depth as the injections at a 10° angle. Ferrules were scored prior to implantation and cemented in place using dental cement (Lang Dental).

Following surgery, rats were administered with Rimadyl (5 mg/kg) subcutaneously. Rats also received subcutaneous injections of Rimadyl (5 mg/kg), once daily for 2–3 d following surgery, and cephalexin (15 mg kg^−1^, PO) was administered orally once per day for 7 d postoperatively. Following behavioral testing, rats were perfused with 4% paraformaldehyde, and their brains were extracted. Brains were postfixed for 48 h in 4% paraformaldehyde, cryoprotected in 30% sucrose, and sectioned on a freezing microtome into 40 µm coronal sections. Sections were mounted on Superfrost slides, coverslipped with a 3:1 glycerol:PBS solution, and imaged on an inverted Nikon Eclipse Ti2 Series Microscope with a DS-Fi3 camera (Nikon Instruments) to verify expression and estimate ferrule depth. Only rats with positive expression were included in the analysis.

### Optogenetic stimulation and testing

Following a 7-day recovery period, rats were reintroduced to the testing arena and habituated to patch cables attached to LED lights and a commutator for 3–5 d (Plexon). During habituation, the LED lights were not illuminated. After habituation, rats were randomly assigned to receive either a blue or yellow light over the 10 test sessions such that by the end of testing, all rats had completed five sessions with blue and yellow light. During behavioral sessions, LEDs were triggered to turn on at the start of a trial (i.e., house light on) and turn off after either once reward was delivered or an error was committed. The light pattern was controlled by the Radiant software package (Plexon). Illumination was set to 50 mW and was checked daily before each session using a compact light meter (Thorlabs). LED-on trials randomly occurred on ∼50% of all trials. We used PlexBright LED Optogenetics Modules controlled by PlexBright Dual LED Commutators to deliver light (blue, 465 nm; yellow, 590 nm) via Thorlab's 200 µm core optical fibers.

The optogenetic construct we used has been reliably established in the optogenetics literature (Han and Boyden, 2007; Zhang et al., 2007; Arrenberg et al., 2010; Calu et al., 2013; Stefanik et al., 2013; Kang et al., 2015; Gardner et al., 2017; Gholami Pourbadie and Sayyah, 2018; Hart et al., 2020). Moreover, a recent patch-clamp experiment using the same construct used in the present work showed that yellow light illumination resulted in clear inhibition of excitatory postsynaptic currents with no rebound excitation (de Lima et al., 2022). Importantly, during all behavioral sessions, LED light was shielded with an electrical tape wrapped around the connecting sleeves and patch cable to minimize the possibility of light distraction. Further, given our experimental design, if light was distracting, we would expect to see effects during both yellow and blue sessions (Gardner et al., 2017), which we see no evidence of.

### Experimental design and statistical analysis

Behavior files were analyzed either using custom-written code in MATLAB (MathWorks 2023b), R (https://www.*R*-project.org/), or GraphPad Prism 10.1 software (GraphPad Software). Percent correct (PC) scores were calculated by dividing the number of correct GO and STOP trials by the total number of trials. RT values were generated by calculating the time from the center port exit to the lever press. Total trials and total reward trials were computed as well. Similar calculations were made after filtering the data for sequence effects (i.e., when a GO preceded a STOP or GO or when a stop preceded either a STOP or GO) as previously published (Bryden et al., 2012, 2016, 2019; Bryden and Roesch, 2015; Tennyson et al., 2018; Brockett et al., 2020a,b, 2022). Planned *t* tests were conducted, where appropriate to verify the directionality of interactions. Unless otherwise specified, all behavioral data (i.e., PC or RT data) was analyzed using a two-way repeated measures ANOVA, where each datum is a session average.

## Results

### Impact of optogenetic inhibition of OFC over all trials

Following completion of training on the stop-change task (Fig. 1*A*), all rats underwent stereotaxic viral injection and ferrule implantation surgery. An inhibitory opsin (rAAV5-CaMKIIa-eNPHR3.0-EYFP-WPRE-PA) was bilaterally expressed throughout the OFC (Fig. 1*B*). Contours mapping the expression for each rat included in the analysis are shown in Figure 1*B*, along with lines demarcating fiber depths. Only rats with EYFP expression were included in these analyses. Rats were allowed 1 week to recover before resuming testing on the stop-change task outlined in Figure 1*A*. In brief, each trial began with illumination of the house light that instructed the rat to make a nose poke into the center fluid well. On 80% of trials, GO trials, nose poking led to the illumination of the left or right light, which signaled the corresponding lever rats needed to press to get reward. On the remaining 20% of trials, STOP trials, nose poking led to the illumination of either the left or right light, which was then extinguished, and the light over the opposite lever was illuminated. On STOP trials, rats were required to stop the movement signaled by the first light and respond in the direction of the second light to receive rewards. GO and STOP trials were randomly interleaved, and trials were presented in a pseudorandom sequence such that left and right trials were presented in equal numbers. On 50% of all trials, an LED light, either blue (control) or yellow (inhibitory), would be illuminated for the duration of the trial (house light on until reward delivery or error commission). The light color was changed in a randomized manner across days in a manner consistent with previous work (Wikenheiser et al., 2017), such that rats underwent 10 total sessions (five, blue; five, yellow). PC and RTs (i.e., time from lever press until fluid well entry) were calculated across session averages.

**Figure 2.**
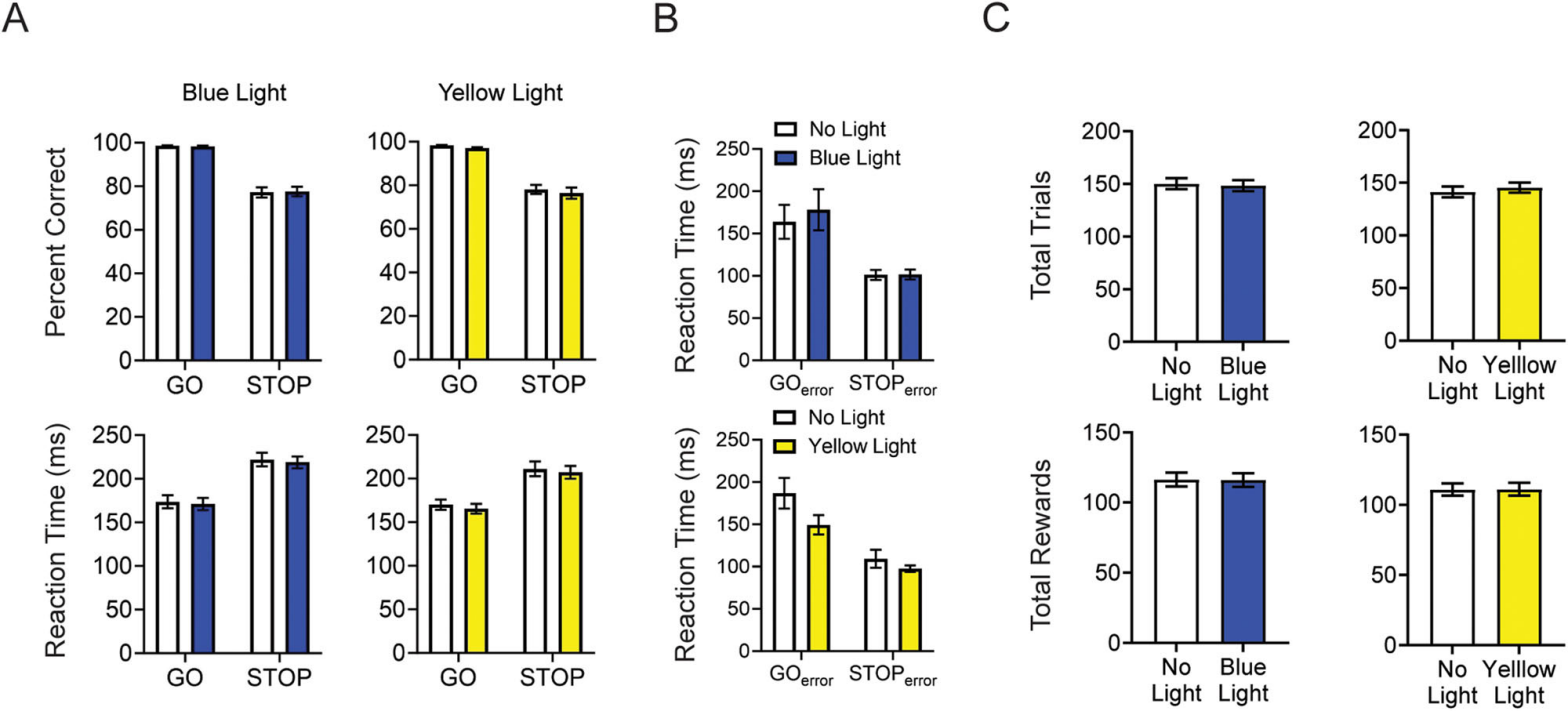
Analysis of stop-change task performance on blue light and yellow light sessions. ***A***, PC and RTs’ GO and STOP trials for blue and yellow light sessions. PC and RTs were averaged over sessions. Error bars represent ±SEM. ***B***, RTs’ GO and STOP error trials for blue and yellow light sessions. RTs were averaged over sessions. Error bars represent ±SEM. ***C***, Counts of the total number of trials and total rewards received for blue and yellow light sessions. Error bars represent ±SEM.

To control the impact of LED illumination on stop-change performance, we first conducted a three-way repeated measures ANOVA (trial type × light × session) on PC and RT data. We observed no main effect of session on either PC [*F*_(1,6)_ = 0.684; *p* = 0.440] or RT [*F*_(1,6)_ = 1.957; *p* = 0.211] data, suggesting that LED illumination alone did not alter performance across test days. Therefore, we collapsed across session and performed a two-way repeated measures ANOVA (trial type × light) in the following analyses. Two-way repeated measures ANOVA revealed a significant main effect of trial type for both PC [*F*_(1,34)_ = 103.5; *p* < 0.0001] and RTs [*F*_(1,34)_ = 67.23; *p* < 0.0001], consistent with STOP trials being more difficult than GO trials. However, there was no main effect of blue light illumination for either PC or RT [PC: *F*_(1,34)_ = 0.0049, *p* = 0.9445; RT: *F*_(1,34)_ = 0.9456, *p* = 0.3377], suggesting that LED illumination on its own did not distract or alter performance (Fig. 2*A*). We also observed no significant interaction [trial type × light; PC: *F*_(1,34)_ = 0.1556, *p* = 0.6957; RT: *F*_(1,34)_ = 0.9456, *p* = 0.3377; Fig. 2*A*]. We also examined RTs on error trials. Because overall task performance was so high, on a subset of trials, no errors were committed on either GO or STOP trials. Therefore, to analyze these data, we performed a mixed-effects analysis with random intercepts to account for the repeated nature of the task design. Blue light illumination did not impact RTs on error trials with only a main effect of trial type being observed [*F*_(1,34)_ = 26.90; *p* < 0.0001; Fig. 2*B*]. Finally, we conducted paired *t* tests and found that blue light LED illumination did not significantly impact the total number of trials completed [*t*_(34)_ = 0.7863; *p* = 0.4372] or the total number of rewards received [*t*_(34)_ = 0.5035; *p* = 0.6179] in a session (Fig. 2*C*).

To examine the impact of yellow light inhibition on OFC function, we performed a three-way repeated measures ANOVA (trial type × light × session) on PC and RT data. We observed no main effect of session for either measure [PC: *F*_(1,6)_ = 0.637, *p* = 0.4550; RT: *F*_(1,6)_ = 1.271, *p* = 0.3030]. As before, we then collapsed across sessions and conducted a series of two-way ANOVAs for PC and RT measures. A two-way repeated measures ANOVA revealed a significant main effect of trial type for both PC [*F*_(1,34)_ = 93.73; *p* < 0.0001] and RTs [*F*_(1,34)_ = 69.12; *p* < 0.0001], consistent with STOP trials being more difficult than GO trials (Fig. 2*A*). However, there was no main effect of yellow light illumination for either measure [PC: *F*_(1,34)_ = 1.630, *p* = 0.2103; RT: *F*_(1,34)_ = 0.7995, *p* = 0.3775], suggesting that OFC inhibition did not alter overall stop-change performance. We also observed no significant interaction [trial type × light; PC: *F*_(1,34)_ = 0.0464, *p* = 0.8306; RT: *F*_(1,34)_ = 0.0049, *p* = 0.9445; Fig. 2*A*]. As with our blue light analysis, we also examined RTs on errors using a mixed-effects model with random intercepts to account for the fact that on some sessions, rats did not commit an error on either GO or STOP trials (Fig. 2*B*). On yellow light days, we observed a main effect of both trial type [*F*_(1,34)_ = 34.33; *p* < 0.0001] and light [*F*_(1,34)_ = 5.247; *p* = 0.0283], but no interaction [*F*_(1,14)_ = 1.458; *p* = 0.2473], suggesting the yellow light illumination had an overall speeding effect on both GO and STOP error trials. Paired *t* tests revealed that OFC inhibition did not result in a significant change in the total number of trials completed [*t*_(34)_ = 1.691; *p* = 0.1001] or the total number of rewards received [*t*_(34)_ = 0.2433; *p* = 0.8093] in a session, suggesting that OFC inhibition did not impact a rat's overall motivation to perform the task (Fig. 2*C*). Thus, when examining the behavior overall on GO and STOP trials, OFC inhibition did not impact PC or RTs on correct trials but did increase the speed on errant trials.

### Optogenetic inhibition of OFC reduces accuracy on difficult STOP trials

Previous work has suggested that single units in the OFC differentially encode STOP trials depending on the identity of the trial type that preceded it (i.e., GO or STOP; Bryden and Roesch, 2015). Further, neural signals representing accurate response direction during performance of STOP-change tasks have been shown to be weaker and more slowly resolved on STOP trials that follow GO trials, reflecting the higher conflict between the two actions (Bryden et al., 2012; Brockett et al., 2020b, 2022). Given this, we hypothesized that OFC inhibition might impact behavior differently broken down by trial sequence (Bryden et al., 2012, 2016, 2019; Bryden and Roesch, 2015; Tennyson et al., 2018; Brockett et al., 2020a,b, 2022). To test this hypothesis, we examined gG (g: previous trial GO; G current trial GO), sG (s: previous trial STOP; G current trial GO), and gS (g: previous trial GO; G current trial STOP) trials. Given the relatively low frequency of STOP trials sS (s: previous trial STOP; S current trial STOP), sS trials will be analyzed separately in the next section due to the infrequency of sS preventing direction comparison over all sessions. Lastly, to determine if the OFC influenced the behavior via mechanisms that were engaged proactively based on past experience or reactivity during the current trial, we assessed whether current (i.e., capital G or S) or previous (i.e., lowercase g or S) illumination impacted performance separately. As above, we conducted two-way ANOVAs separately for blue and yellow light sessions.

**Figure 3.**
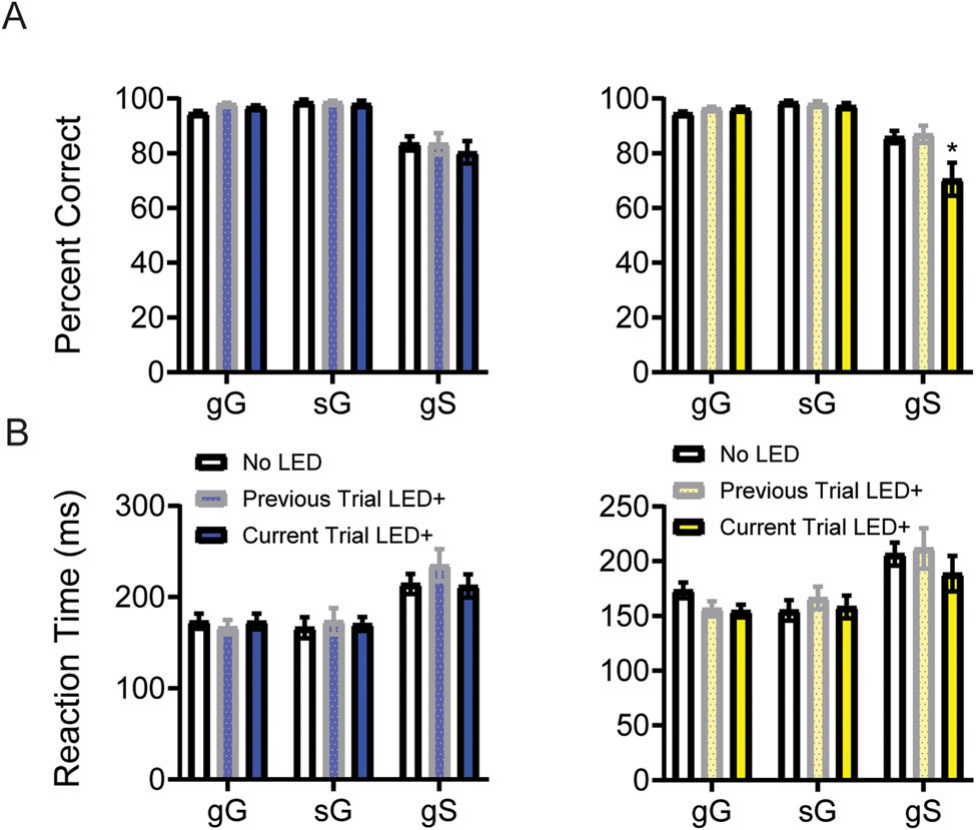
Analysis of trial-level sequence effects on blue and yellow light days. PC (***A***) and RTs (***B***) for sequence effects for blue and yellow light sessions. PC and RTs were averaged over blue and yellow light sessions, respectively. Gray bars indicate trials where LED illumination occurred on the preceding trial (lowercase letter). Full color bars indicate trials where LED illumination occurred on the current trial (uppercase letter). Error bars represent ±SEM. Trial types represented as follows: gG, go, Go; sG, stop, Go; gS, go, Stop.

Consistent with the blue light findings presented in Figure 3, blue light illumination did not alter accuracy (PC) or reaction speed (RT) regardless of trial sequence. A mixed-effects model with random intercepts revealed a main effect of trial type [PC: *F*_(2,68)_ = 40.27, *p* < 0.0001; RT: *F*_(2,68)_ = 22.46, *p* < 0.0001], but no effect for light [PC: *F*_(2,68)_ = 0.4708, *p* = 0.6265; RT: *F*_(2,68)_ = 0.4379, *p* = 0.6472] or a significant interaction [PC: *F*_(4,135)_ = 0.6421, *p* = 0.6334; RT: *F*_(4,134)_ = 0.9716, *p* = 0.4253; Fig. 3*A*].

To investigate whether OFC inhibition altered accuracy and RTs, we performed the same analysis on yellow light data. For performance accuracy, we observed a significant main effect for trial type [*F*_(2,68)_ = 35.55; *p* < 0.0001] and light [*F*_(2,68)_ = 5.799; *p* = 0.0047], as well as a significant interaction (trial type × light) [*F*_(4,132)_ = 6.021; *p* = 0.0002; Fig. 3*B*]. Tukey-corrected pairwise comparisons revealed these effects were driven by a significant decrease in accuracy on gS trials, but only when the yellow light was illuminated on the STOP trial, and not on the previous GO trial (no LED vs current trial LED on: *p* = 0.0001; previous trial LED on vs current trial LED on: *p* = 0.0001; no LED vs previous trial LED on: *p* = 0.9777; Fig. 3*A*). This suggests that on gS trials, where rats must suppress and redirect a habitual GO response, the OFC is necessary to engage mechanisms of inhibitory control. For RT, as before, we observed a significant main effect of trial type [*F*_(2,68)_ = 15.59; *p* < 0.0001] but no significant main effect for light [*F*_(2,68)_ = 1.121; *p* = 0.3319] or a significant interaction [*F*_(4,129)_ = 0.7561; *p* = 0.5558; Fig. 3*B*].

### OFC inhibition does not appear to alter behavior on sS trials

**Figure 4.**
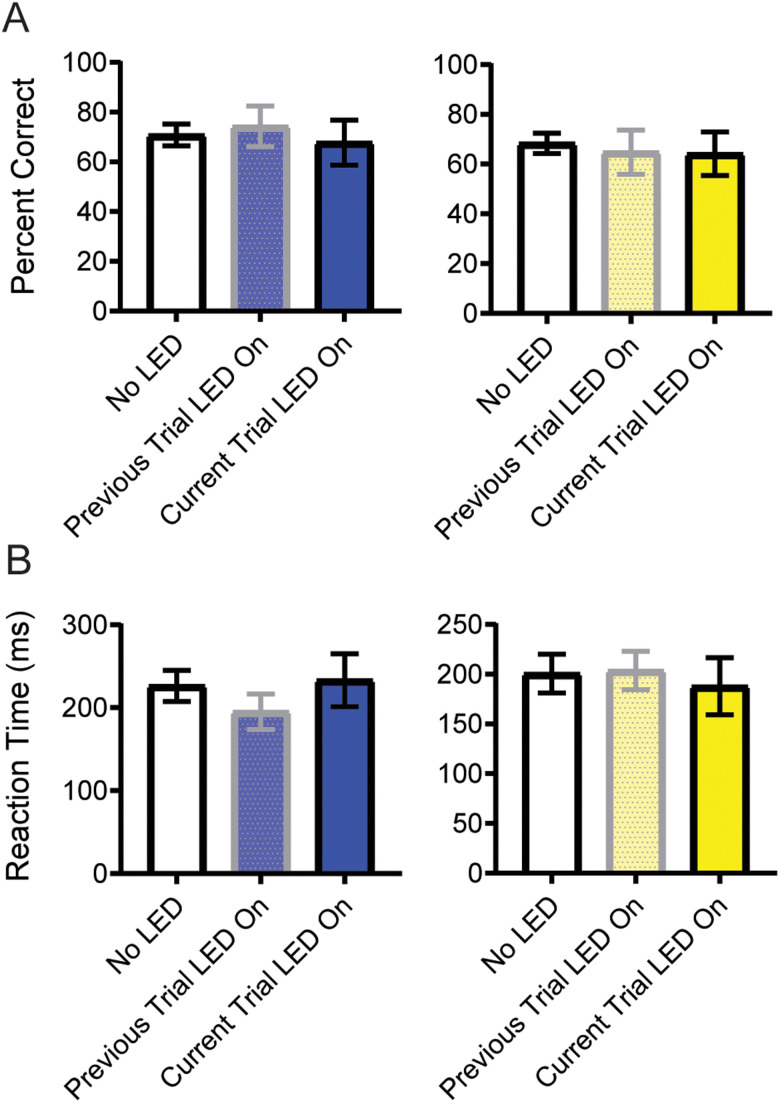
Analysis of trial-level sS performance on blue and yellow light days. PC (***A***) and RTs (***B***) for sS (stop, Stop) trials for blue and yellow light sessions. Gray bars indicate trials where LED illumination occurred on the preceding trial (lowercase letter). Full color bars indicate trials where LED illumination occurred on the current trial (uppercase letter). Error bars represent ±SEM.

We have shown that OFC inhibition selectively disrupts performance on gS trials, only when the inhibition occurs on the STOP trial (i.e., the trial that requires inhibitory control), but not on the preceding trial. While a complete dataset including sS trials was not possible given the relatively few instances of sS trials in combination with even fewer sS trials that could be divided among the three possible light conditions (i.e., no LED, previous trial LED, current trial LED), we still wanted to determine whether there was any impact of OFC inhibition on sS trials. To address this issue, we examined only sessions that had at least one of each trial type in both directions (*n* = 23 yellow sessions and *n* = 23 blue sessions). We performed a mixed-effects analysis with random intercepts and observed no significant impact of blue light exposure on sS trial accuracy [*F*_(2,37)_ = 0.2246; *p* = 0.7999] or RT [*F*_(2,28)_ = 0.680; *p* = 0.3916], consistent with all of our other findings that LED illumination on its own does not impact performance of the stop-change task (Fig. 4*A,B*). We performed a separate one-way ANOVA on accuracy and RT data on yellow light sessions as well. We observed no significant impact of OFC inhibition on accuracy [*F*_(2,57)_ = 0.0967; *p* = 0.9080] or RT [*F*_(2,26)_ = 0.0075; *p* = 0.9925]. While not without caveat given the relatively few sessions, these findings suggest that OFC inhibition does not impair sS performance.

## Discussion

In this study, we optogenetically inhibited the OFC in rats performing a stop-change task. While overall, OFC inhibition did not reduce stop-change performance, optogenetic inhibition did speed overall RTs on error trials, and analysis of trial sequence revealed a significant impairment of inhibitory control. This impairment was selective, in that on STOP trials where the animal had previously been rewarded for performing a GO response, optogenetic inhibition disrupted the recruitment of inhibitory control processes, thereby reducing accuracy on these gS trials. Critically, these results hold regardless of whether we compare accuracy on gS trials in which no light was present or gS trials where OFC inhibition occurred on the preceding GO trial only. These findings suggest that on the stop-change task, a task where reward value is held constant, impairment of OFC function negatively impacts mechanisms supporting the instantiation of inhibitory control as evidenced by increased gS errors and fast inappropriate responding on error trials. Previous work using the stop-change task has shown that the dorsal medial striatum (DMS) supports stop-change trial performance by signaling actions to both the GO and STOP-change cue (Bryden et al., 2012). Unilateral excitotoxic lesions to the anterior cingulate cortex (ACC) disrupt and delay the ability of DMS to represent both intended actions, ultimately resulting in impaired performance on STOP-change trials (Brockett et al., 2020b). Previous work has suggested that striatal inputs to the OFC contribute to inhibitory control processes; therefore, it would be interesting for future work to compare the contributions of the OFC relative to the ACC to stop-change performance.

While early case study and OFC lesion studies suggested a role for the OFC in inhibitory control, the last few decades have seen that the models of OFC function shift away from inhibitory control and more in the direction of either value-guided decision-making or toward the maintenance of cognitive maps (Rudebeck and Rich, 2018; Brockett and Roesch, 2021; Gardner and Schoenbaum, 2021; Knudsen and Wallis, 2022; Shi et al., 2023). While roles for the OFC in inhibitory control, value-guided decision-making, and abstract representations of cognitive space are not mutually exclusive of each other, the latter two accounts have garnered much support in recent years with single-unit studies using increasingly complex reward-based decision-making tasks across multiple species showing that the OFC robustly tracks decision variables such as values and outcomes as well as mapping these features to task representations (Gottfried et al., 2003; Wallis and Miller, 2003; Padoa-Schioppa and Assad, 2006; Hare et al., 2008; Rich and Wallis, 2016; Schuck et al., 2016; Hirokawa et al., 2019; Zhou et al., 2019, 2021; Kuwabara et al., 2020; Costa et al., 2023).

Despite this focus on the OFC in complex value-based decision-making, several studies in rodents (Chudasama and Robbins, 2003; Eagle et al., 2008; Bryden and Roesch, 2015), nonhuman primates (Mansouri et al., 2014; Balasubramani et al., 2020), and humans (Solbakk et al., 2014) have suggested a consistent role for the OFC in inhibitory control processes as well. Importantly, careful reviews of the ever-growing OFC literature have implicated portions of lateral OFC (LO) specifically in inhibitory processes (Jentsch et al., 2014; Izquierdo, 2017). Unlike value-based decision-making tasks where value is often directly manipulated, the primary goal of the inhibitory control tasks, such as the stop-change task, is to test a subject’s ability to inhibit a prepotent or habitual response in the presence of unexpected sensory information (Verbruggen et al., 2019). These tasks, necessarily, keep reward value constant and reward subjects the same amount for correct responses (Verbruggen et al., 2019). Single-unit studies have shown that neurons in the OFC differentiate between GO and STOP trials and are modulated by the identity of the previous trial type (i.e., GO or STOP; Bryden and Roesch, 2015). The current work partially supports these findings, in that optogenetic inhibition of the OFC during gS trials resulted in impaired performance, although we did not observe similar effects for sS trials in this study. This difficulty in assessing sS trials was due in part to the relatively infrequent nature of STOP-change trials, a feature of most stop-change and stop-signal tasks, but also due the parcellation of data into no LED, previous LED, and current LED trials. This limits our ability to generalize our effects to all STOP-change trials, though we would predict that with adequate number of trials, the differences we see on gS trials might generalize to sS trials as well. Further, in nonhuman primates, single-unit encoding of inhibitory control was found to be orthogonal to value encoding, suggesting that the OFC likely maintains discrete representations of both processes (Balasubramani et al., 2020).

The instantiation of inhibitory control is clearly not the sole function of the OFC; however, as shown here and in the past work, impairment of OFC functioning disrupts inhibitory control, and neurons in the OFC do seem to encode the need for control separately from value. Integrating this information in current models of OFC functioning is needed to more fully understand how the OFC supports behavior. Within models of economic decision-making, inhibitory control signals have been suggested to contribute to action planning, allowing the brain to appropriately weight necessary reward information and bias behavioral output accordingly without interference from competing action plans (Yoo and Hayden, 2018). Similarly, within the cognitive map framework, inhibitory control may be essential for behavioral flexibility, allowing the transition between task states as reward contingencies, the sensory environment, or overall context changes (Brockett and Roesch, 2021). Further work is needed to better understand how and what neurons in the OFC integrate inhibitory control information with decision variables. It is also possible that the results of our manipulation might also reflect a deficit in the redirection of correct response STOP trials. While we cannot rule out this possibility completely, the fact that rats, regardless of OFC inhibition, are significantly faster when committing errors on STOP-change trials (Fig. 2*B*) and are not slower on correct STOP-change trials suggests that rats are responding reflexively to the first cue before it is possible to engage mechanisms to redirect the movement. Nevertheless, this is an average measure (i.e., reflects both inhibiting and redirecting); thus, it is possible rats might have also been impaired in redirection behavior on STOP trials.

The present study attempted to establish a causal role for the OFC in inhibitory control processes and showed that OFC inhibition speeds error commission and reduces accuracy on trials requiring inhibitory control the most. The OFC receives inputs from numerous other brain regions including sensory and insular cortices as well as striatum, limbic, and thalamic structures (Jentsch et al., 2014; Izquierdo, 2017; Shi et al., 2023). This functional heterogeneity makes careful regional and cell-type dissection of the OFC critical developing theoretical explanations of OFC involvement (Izquierdo, 2017). It is likely that these varied inputs interact with multiple local circuits. While in this study, only excitatory neurons were inhibited mostly in LO, it would be interesting to dissect the contributions of other neuronal cell types in this area to inhibitory control processing and the integration of value and control signals.

## Synthesis

Reviewing Editor: Sam Golden, The University of Washington Decisions are customarily a result of the Reviewing Editor and the peer reviewers coming together and discussing their recommendations until a consensus is reached. When revisions are invited, a fact-based synthesis statement explaining their decision and outlining what is needed to prepare a revision will be listed below. The following reviewer(s) agreed to reveal their identity: Alicia Izquierdo.

Dear Dr. Brockett,

Your manuscript has been read by two experts. Overall, the reviewers found the topic of the manuscript to be of interest to the field, and the content to be potentially appropriate for publication if significant edits are provided. After discussion, the reviewers concluded that significant revision and re-view are necessary. Below I provide a brief synthesis of the reviewer comments, followed by the comments themselves. With any resubmission, please include a point by point rebuttal of both Reviewer's specific comments.

Synthesis: The manuscript examines the role of the orbitofrontal cortex (OFC) in inhibitory control through a stop-change task in rats, with a focus on the effects of OFC inhibition on specific trial types. Reviewer 1 highlights the manuscript's innovative approach to revisiting the OFC's role in inhibitory control, contrasting it with the prevalent focus on its involvement in value-based decision-making. They point out the nuanced and subtle effect of OFC inhibition, questioning the clarity of its role in inhibitory control, and call for a deeper exploration of the OFC's neuroanatomical heterogeneity and its implications. The omission of key literature and the choice of experimental tasks are also noted as areas for improvement. Reviewer 2 raises critical concerns regarding the experimental validation and statistical analysis. They emphasize the lack of verification for the optogenetic manipulation, inappropriate statistical methods for the repeated measures design, and questionable subgroup analysis that could lead to misleading conclusions. Both reviewers suggest that while the study addresses a significant question, major issues with experimental validation, statistical analysis, and theoretical framing need to be addressed to substantiate the findings and clarify the OFC's role in inhibitory control.

Reviewer 1:

Authors report results from a stop-change task to test whether OFC is involved in inhibitory control, which is in fact an older account of its function. Authors expressed halorhodopsin in excitatory neurons in OFC and found that activation of those neurons significantly decreased accuracy only on STOP trials that followed GO trials (gS), not STOP trials preceded by a STOP trial (sS). Inhibition also speeded reaction times on error trials. The results are discussed in relation to existing OFC literature that has instead emphasized its role in value-based decision making and (more) cognitive functions.

The effect reported here is small and subtle- OFC inhibition did not reduce stop-change performance, but did speed overall reaction times on errors, and on gS trials. I am not sure that this is a "clear role for the OFC in inhibitory control."

I agree completely that the historical account of OFC in inhibitory control has been neglected and ignored in favor of the more "modern" cognitive accounts mentioned above. Authors injected eNpHR3.0 in what appears to be mostly ventrolateral OFC. As there is documented functional heterogeneity within OFC (Izquierdo, 2017), is it possible had authors placed the optic fiber more laterally, there would not have been such modulation of gS? I would appreciate a discussion of the neuroanatomical connectivity that would render parts of OFC more 'motor-control' connected than others?

Related to inhibitory control, the comprehensive, highly relevant work by Jentsch and colleagues on impulsivity is not cited (Jentsch et al. 2014, Ann N Y Acad Sci, see Figure 1).

Does optogenetic inhibition of pyramidal neurons in OFC affect performance on a stop signal reaction time task, a canonical test of response inhibition? Or in a go-no go task? I wonder why those classic tasks were not used to probe this question and instead this stop-change task.

"Rats were trained on the stop-change task for approximately 1 month before undergoing virus injection and ferrule implantation surgery" Could results have differed had OFC been instead inactivated during initial training rather than after extensive training? Also, it would be nice to see training data- there are no details about this phase.

Minor

There are no titles to Figures 2-4.

A point of clarification in the review of the literature- though large aspiration lesions of OFC produce deficits in reversal learning in macaques (Izquierdo et al. 2004), selective excitotoxic lesions of OFC do not (Rudebeck et al. Nat Neurosci, 2013). Additionally, the degree to which reversal learning accounts for 'inhibitory control' may depend on task requirements- and could be discussed further.

Reviewer 2:

Authors (Required) In this manuscript, the authors aimed to causally test the role of rat OFC in inhibitory control. They report an effect on a stop-change task that is specific to a subset of trials when the need for inhibitory control should theoretically be the greatest, and conclude that the OFC is involved in inhibiting pre-potent actions.

As the authors note, the role of OFC in behavioral inhibition has a long history of study. Much of the field no longer considers this a major function of OFC, and some argue that the notion of response inhibition is a poor lens through which to consider OFC function. However, probing the role of OFC on this task certainly remains a legitimate experimental question to address. Unfortunately, the combination of inappropriate statistical analyses and a lack of verification of the main experimental manipulation make the data impossible to interpret in its current form.

Major issues:

1) Validation of optogenetic manipulation.

There is no viral expression data shown, nor are any fiber tracts reconstructed to verify that light was delivered to the region where the virus was targeted. Not only is this information not presented, the methods do not mention whether the authors checked these things at all! They cite a study that used patch clamp recordings to verify the efficacy of optogenetic stimulation in vitro; I don't know whether this study was performed in the authors' laboratory, but whether or not it was, it clearly was not performed on the rats that were used in this study, as the cited work was investigating ACC. It is unclear how that citation in any way suggests that the author's manipulations were efficacious. I don't think that anyone doubts that optogenetics *can* work; the question is whether it worked in the way the authors intended it to in this study. Presently there is no particularly strong reason to think it did.

There is often variation in titer between batches of virus, as well as considerable differences in surgical practices between individuals, not to mention a million other tiny details that can affect whether a sophisticated manipulation such as this actually functions. So citing other work doesn't speak to the rats used in this study. Consequently, it is absolutely critical to include example images showing that the construct did indeed express (in the very rats that were used in this study), to report the extent of expression across animals, to verify that fibers were placed in the area of expression, and of course to exclude any animals for which these criteria were not met. This is the absolute minimal acceptable verification of an optogenetic manipulation; this still falls short of directly showing that neurons in these rats were inhibited when the LEDs were turned on. But reporting these data would provide a reasonable basis to think that the stimulation might have been effective.

2) Inappropriate statistical analysis.

The authors are using ANOVA as their main statistical analyses, but their data involve repeated measures, so this approach is not valid. All of the ANOVAs in the manuscript need to be replaced with either repeated measures ANOVAs or mixed-effects models with random intercepts for each subject in order to correctly account for the repeated measures inherent to this experimental design. Treating repeated measures as independent data points drastically increases the likelihood of incorrectly rejecting the null hypothesis in tests like ANOVA. Given this, it's not possible to evaluate any of the statistical conclusions presented in the manuscript.

3) Questionable subgroup analysis.

The authors do report a behavioral effect, which might suggest that their opto manipulation was effective. However, this effect is currently founded on incorrect statistical analysis (see above). Moreover, the effect emerges only when the authors investigate a small subset of trials that occurred in the context of a particular sequence of trial types. Looking only at high-conflict stop trials does not seem have been part of the authors' original experimental intent. (I infer this because if this was a pre-planned experimental approach I assume the authors could have programmed trials such that they would have sufficient numbers of sS examples to analyze as well.) This is a border-line questionable statistical practice that is pretty close to p-hacking; introducing unplanned subgroup comparisons effectively increases the degrees of freedom of the analysis without accounting for in the statistical test, which again inflates the false positive rate of statistical tests. At the same time, it reduces statistical power (or the accuracy of point estimates when session means are compared, as here) by decreasing the sample size.

These things could perhaps be overlooked if there was a strong theoretical reason to suppose these trials should in fact be special. But this does not seem to be the case. I agree that gS trials could theoretically be thought of as more difficult that sS trials, but based on the authors graphs rats evidently do not agree, as percent correct actually appears to be higher for the ostensibly more difficult gS trials (~85% correct) compared to "easier" sS trials (~70% correct; though I acknowledge that the author's ability to measure this number accurately is limited due to the very small number of trials; i.e. <1/session, on average). This effect going the wrong direction to the author's hypothesis notwithstanding, it seems improbable that if the OFC's role it to perform inhibitory control it's not necessary for all stop trials (even though it's clear that stop trials are on average quite a lot more difficult than go trials) and only needs to be engaged in the relatively rare case of extra-difficult high-conflict inhibition cases (which rats' behavior actually suggest are easier than what should be the low-conflict cases).

Other issues

1) There are numerous inconsistencies between what is reported in the methods and main text. The authors state in main text that the frequency of light used in each session was randomized, but the methods state that light frequency alternated in a predictable way from session to session. The methods say that rats were tested for 20 total sessions while the main text says 10 total sessions. The methods say the fibers from Thor Labs were used in the experiment, but the main text refers to fiber implants from plexon.

2) I realize this task has been used previously, but I do not think it provides strong evidence specifically for inhibitory control. The task creates a situation where goals change very quickly on a subset of trials. Incorrect responses are interpreted as resulting from a failure to quickly stop the ongoing action, but they could be explained just as well as a failure to quickly initiate the correct new action. The OFCs role may not be to stop ongoing things, but rather to determine the current goal. Failing to quickly update the current goal isn't the same as failing to inhibit an ongoing action, and I don't see how the task can differentiate these possibilities. This is at least part of the reason why the response inhibition explanation of OFC function has largely fallen out of favor (along with other evidence discussed in some of the papers the authors cite in the intro). At the very least, some discussion should be given over to alternative possible interpretations of this task.

3) Why do the authors characterize OFC as "largely undefined" in the introduction? Do they take issue with any of the parcellations of frontal cortex that have been proposed (in either rats or primates)? If so, they could be more specific because otherwise this statement seems unsupported.

4) Only male rats were tested, so the title should specifically reflect this

5) lines 156-159: I didn't understand the relevance of this statement to the present study. The authors are performing only inhibition (unless I deeply misunderstood something), so why is it relevant that some experiments use both excitation and inhibition?

6) Typos:

line 89: wase --> was

line 97: "lever" --> "...the lever..."

line 116: "a single bilateral injection" sounds self-contradictory (though I understand what the authors mean).
